# Association of Formulary Prior Authorization Policies With Buprenorphine-Naloxone Prescriptions and Hospital and Emergency Department Use Among Medicare Beneficiaries

**DOI:** 10.1001/jamanetworkopen.2020.3132

**Published:** 2020-04-20

**Authors:** Tami L. Mark, William J. Parish, Gary A. Zarkin

**Affiliations:** 1 RTI International, Rockville, Maryland

## Abstract

**Importance:**

Prior authorization requirements may be a barrier to accessing medications for opioid use disorder treatment and may, therefore, be associated with poor health care outcomes.

**Objective:**

To determine the association of prior authorization with use of buprenorphine-naloxone and health care outcomes.

**Design, Setting, and Participants:**

This comparative interrupted time series analysis examined enrollment and insurance claims data from Medicare beneficiaries with an opioid use disorder diagnosis or who filled a prescription for an opioid use disorder medication between 2012 and 2017. Over this period, 775 874 members were in 1479 Part D plans that always required prior authorization, 113 286 members were in 206 plans that removed prior authorization, 189 461 members were in 489 plans that never required prior authorization, and 619 919 members were in 485 plans that added prior authorization. Data analysis was performed from April 2019 to February 2020.

**Exposures:**

Removal or addition of prior authorization and new prescriptions filled for buprenorphine-naloxone.

**Main Outcomes and Measures:**

Buprenorphine-naloxone use, inpatient admissions, emergency department visits, and prescription drug and medical expenditures.

**Results:**

The study population in 2012 included 949 206 Medicare beneficiaries (mean [SD] age, 57 [15] years; 550 445 women [58%]). Removal of prior authorization was associated with an increase of 17.9 prescriptions (95% CI, 1.1 to 34.7 prescriptions) filled for buprenorphine-naloxone per plan per year, which is a doubling of the number of prescriptions, on average. Each prescription filled was associated with statistically significant decreases in adverse health care outcomes: substance use disorder–related inpatient admissions decreased by 0.1 admission per plan per year (95% CI, −0.2 to −0.1 admission per plan per year), and substance use disorder–related emergency department visits decreased by 0.1 visit per plan per year (95% CI, −0.13 to −0.03 visit per plan per year) (all *P* < .001). Combining these results, removal of prior authorization was associated with a reduction in substance use disorder–related inpatient admissions by 2.0 admissions per plan per year (95% CI, −4.3 to −0.1 admissions per plan per year) and substance use disorder–related emergency department visits by 1.4 visits per plan per year (95% CI, −3.2 to −0.1 visits per plan per year).

**Conclusions and Relevance:**

Removing prior authorization for buprenorphine-naloxone was associated with an increase in the medication use and decreases in health care utilization and expenditures.

## Key Points

**Question:**

Is prior authorization associated with reduced use of buprenorphine-naloxone and increased emergency department visits and hospitalizations?

**Findings:**

This comparative interrupted time series analysis of Medicare beneficiaries with opioid use disorders found that Medicare Part D plans that removed prior authorization had an associated increase in the use of buprenorphine-naloxone, whereas plans that added prior authorization had an associated decrease in buprenorphine-naloxone use. Higher rates of buprenorphine-naloxone use were associated with lower emergency department visits and hospitalizations.

**Meaning:**

These findings suggest that policies to remove prior authorization on buprenorphine-naloxone may be associated with increased use of these medications and improved health care outcomes.

## Teaser

This study uses a comparative interrupted time series analysis to examine the association of prior authorization with use of buprenorphine-naloxone and health care outcomes among Medicare beneficiaries.

## Introduction

Buprenorphine is an effective medication for treating opioid use disorders (OUDs). It can substantially reduce the risk of mortality and facilitate long-term recovery.^1,2,3,4,5,6^ Studies show that patients who receive buprenorphine have reduced emergency department (ED) and inpatient use compared with those who do not receive OUD medications,^7,8,9^ that longer time taking buprenorphine is associated with reduced ED use,^10,11,12,13^ and that expansion of the number of institutions and clinicians providing buprenorphine in a region is associated with reduced ED and inpatient use.^14^

Federal and state governments are focused on improving access to OUD medications by training more professionals to prescribe them; expanding the delivery of OUD treatment in specialty addiction programs, hospitals, community health centers, and criminal justice settings; and encouraging coverage of OUD medications in Medicaid, Medicare, and private insurance programs. Because prior authorization has been shown to reduce access to medications, some public and private programs have removed prior authorization requirements for OUD medications.^15,16^ However, prior authorization for buprenorphine is still common under Medicaid and private insurance.^17,18,19^

Medicare Part D offers a unique opportunity to study the association of changes in prior authorization policies with the use of OUD medications and, more broadly, with health care outcomes. Medicare Part D benefits are provided by private health plans or by Medicare Advantage prescription drug plans. Each plan determines its own coverage policies, such as which medications are subject to prior authorization and which medications are included on the formulary, within parameters set by Centers for Medicare & Medicaid Services. Information on the use of prior authorization by medication within each Medicare Part D formulary is available to the public in a central database. Thus, researchers can study the association between variations in use of prior authorization across a large population and health care outcomes.

Understanding how to improve OUD treatment access and outcomes among Medicare beneficiaries is important. The number of older Medicare beneficiaries and those with physical disabilities with an OUD is large and growing.^20,21^ In 2016, 1.5% of the approximately 60 million Medicare beneficiaries were diagnosed with an OUD.^22^ Between 2007 and 2016, 26% of older Medicare beneficiaries were using prescription opioids, and 52% of Medicare beneficiaries with physical disabilities were using prescription opioids.^23^ Among adults aged 65 and older, ED visits for opioid misuse tripled between 2006 and 2014,^24^ and between 2010 and 2015, opioid-related inpatient admissions increased by 54.4%.^25^ Although, there has been a documented increase in the number of older adults seeking treatment for OUD, only 27% of Medicare beneficiaries with OUD diagnosis received a medication to treat their OUD.^20,21^

This study aimed to determine the association of prior authorization for buprenorphine-naloxone with the rate of use of buprenorphine-naloxone, and the association of prior authorization with inpatient admissions, ED visits, and health care costs. To answer these questions, we use insurance claims and enrollment data from Medicare beneficiaries with OUD who were enrolled in Part D plans.

## Methods

### Study Population and Data Source

The RTI institutional review board reviewed the research project and determined it does not constitute research with human participants because it used deidentified data. Thus, the need for informed consent was waived.

Data were obtained from the Medicare Fee-for-Service claim and enrollment files (Inpatient, Outpatient, SNF, Carrier, and Master Beneficiary Summary Files), as well as the Part D Event, Drug Characteristics, and Formulary files for 2012 to 2017. We requested that the Centers for Medicare & Medicaid Services Research Data Assistance Center provide data on any Medicare beneficiary who had a primary or secondary OUD diagnosis at any time between 2012 and 2017 or who had filled a prescription or had a claim for an OUD medication (ie, either filled prescriptions for buprenorphine or buprenorphine-naloxone, or had a methadone procedure code) over the same time period. As shown in the Figure, there were 1.59 million beneficiaries included who met these criteria, and these beneficiaries were enrolled in one of 6612 unique Part D plans in the marketplace between 2012 and 2017. We subsequently excluded person-months where individuals were not enrolled in Medicare Parts A, B, and D during the month (resulting in 6612 plans and 1.40 million beneficiaries), and we excluded Part D plans where there were fewer than 20 enrollees with OUD (resulting in 2735 plans and 1.38 million beneficiaries). Finally, to isolate the impact of removing or adding prior authorization requirements for buprenorphine-naloxone medications, we split the analysis sample into 2 groups. The first group included plans that always required prior authorization (1479 plans and 775 874 beneficiaries) and plans that removed a prior authorization requirement for these medications (206 plans and 113 286 beneficiaries). The second group included plans that never required prior authorization (489 plans and 189 461 beneficiaries) and plans that added a prior authorization requirement for these medications (485 plans and 619 919 beneficiaries). Race/ethnicity information was obtained from the Medicare Beneficiary Summary File. Persons were classified as either white or nonwhite.

**Figure.**
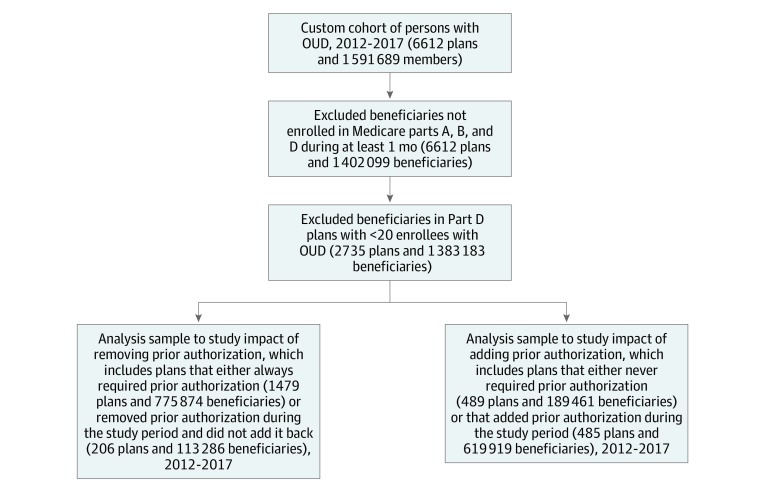
Exclusion Criteria and Final Sample Sizes OUD indicates opioid use disorder.

### Prior Authorization for Buprenorphine-Naloxone Medications

Information on prior authorization was obtained from the Part D formulary files from 2012 to 2017. These files contain a complete list of medications that are covered by each Part D plan and whether they require prior authorization. We coded plans as requiring prior authorization if they required prior authorization on all their covered buprenorphine-naloxone products and coded plans as not requiring prior authorization if they did not require prior authorization on at least 1 of their covered buprenorphine-naloxone products. We chose not to look at buprenorphine products without naloxone because buprenorphine-naloxone is used much more frequently than buprenorphine monoproducts and because buprenorphine monoproducts may have a higher abuse potential, which could influence a Part D plan’s decisions to require or not require prior authorizations for these products.^26^ More information on the coverage of buprenorphine-naloxone products during the study period is available in eTable 1 in the Supplement.

### Outcomes

The use of buprenorphine-naloxone was measured as the number of persons with OUD in each plan and month who filled a new prescription for buprenorphine-naloxone, as well as the total number of buprenorphine-naloxone fills, which includes both new prescriptions and refills. Persons were considered as filling a new prescription if they had not filled a prescription for buprenorphine-naloxone in either of the previous 2 months.

Health care utilization and costs were measured as the number of all-cause and substance use disorder (SUD)–related inpatient admissions, all-cause and SUD-related ED visits, prescription drug expenditures, and nondrug medical care expenditures. Encounters and expenditures associated with SUD were identified as such if the claim included a primary or secondary SUD diagnosis. All variables were summarized at the Part D plan-month level (eg, health care expenditures were calculated as the mean per member per month expenditures among each plan’s OUD population).

### Statistical Analysis

All regression analyses were estimated using data at the plan-month level. We chose to model the data at the plan-month level, as opposed to at a beneficiary level, because the main exposure variable primarily varies at the plan level, with rare exceptions occurring when beneficiaries switch to a new plan from 1 year to the next. Moreover, modeling at the plan-month level allows our results to better inform policy pertaining to Part D plans.

To determine whether removing (or adding) prior authorization from (or to) formularies is associated with an increase (or decrease) in the use of buprenorphine-naloxone, we estimated a comparative interrupted time series analysis using a generalized linear model. The model used a log link and assumed Poisson errors. To account for different sample sizes across plan-month observations, we used the number of persons in each plan and month as an offset term. Beyond the parameters of the comparative interrupted time series specification, the model also controlled for a set of plan-level demographic characteristics, which include mean age, percentage female, percentage who were dually eligible for Medicare and Medicaid, and percentage who were entitled for Medicare because of disability.

To determine the association between prior authorization and changes in health care outcomes, we used a generalized linear model to measure the association between the number of persons filling new buprenorphine-naloxone prescriptions and the number of persons with inpatient admissions, the number of persons with ED visits, and health care spending. Except for health care spending, these models used a log link and assumed Poisson errors. The health care spending models used a log link and assumed γ errors. These models also controlled for the number of persons in each plan and month by including an offset term and controlled for the same plan-level demographic characteristics as described already.

We then used mediation analysis to determine the association between removing prior authorization and health care outcomes.^27,28,29^ The basic approach was to multiplicatively combine the result (regression coefficients) from the analysis measuring the association between prior authorization and the use of buprenorphine-naloxone with the result (regression coefficients) from the analysis measuring the association between buprenorphine-naloxone use and health care outcomes. This provides a measure of the extent to which removing prior authorization may improve health care outcomes by increasing use of buprenorphine-naloxone. To calculate SEs, 95% CIs, and *P* values, we used the suest command in Stata statistical software version 15 (StataCorp) to construct a joint variance matrix across both models.

All the models were estimated using generalized linear models in Stata 15. All hypotheses were tested using 2-sided tests of statistical significance with a threshold of *P* < .05. Data analysis was performed from April 2019 to February 2020.

## Results

Table 1 shows that in 2012, the study population included 949 206 Medicare beneficiaries (mean [SD] age, 57 [15] years); 550 445 (58%) were female, 223 081 (24%) were nonwhite, 616 906 (65%) had physical disabilities, and 544 666 (57%) were dually eligible for Medicare and Medicaid. Additionally, in 2012, 15% of the study population (145 980 beneficiaries) had a medical encounter with a non-OUD SUD primary or secondary diagnosis, and 19% of the study population (179 466 beneficiaries) had a medical encounter with an OUD primary or secondary diagnosis. During this same year, 8% of the population (78 061 beneficiaries) filled new buprenorphine-naloxone prescriptions, and 13% (118 875 beneficiaries) filled any buprenorphine-naloxone prescriptions. Moreover, 28% of the population (270 430 beneficiaries) had any inpatient admissions, 10% (98 906 beneficiaries) had an SUD-related inpatient admission, 46% (434 866 beneficiaries) had any ED visits, and 7% (70 004 beneficiaries) had an SUD-related ED visit. The mean (SD) prescription drug expenditure was $505 ($911) per month, and the mean (SD) nondrug medical care expenditure was $1133 ($2707) per month. Table 1 also shows a breakdown of study population characteristics by the following 4 groups: persons in plans that always required prior authorization, persons in plans that removed prior authorization, persons in plans that never required prior authorization, and persons in plans that added prior authorization during the study period. The characteristics of these 4 groups were not substantially different.

**Table 1.** Population Description in 2012

Characteristic	Beneficiaries, No. (%)				
	All plans (N = 949 206)	Plans that always required PA (n = 432 263)	Plans that removed PA (n = 17 786)	Plans that never required PA (n = 68 595)	Plans that added PA (n = 272 430)
Beneficiary characteristics					
Age, y					
Mean (SD)	57 (15)	56 (14)	62 (14)	58 (15)	60 (15)
18-24	7482 (1)	3841 (1)	32 (0.2)	469 (1)	1831 (1)
25-34	62 705 (7)	31 582 (7)	677 (4)	4127 (6)	14 188 (5)
35-44	120 443 (13)	60 424 (14)	1601 (9)	8217 (12)	28 271 (10)
45-54	224 726 (24)	111 186 (26)	3193 (18)	15 394 (22)	55 033 (20)
55-64	205 342 (22)	97 059 (22)	3446 (19)	14 686 (21)	54 509 (20)
65-74	216 288 (23)	87 026 (20)	5462 (31)	17 067 (25)	75 452 (28)
75-84	88 188 (9)	32 848 (8)	2692 (15)	6754 (10)	33 559 (12)
≥85	24 031 (3)	8297 (2)	683 (4)	1881 (3)	9587 (4)
Female	550 445 (58)	247 822 (57)	10 733 (60)	40 138 (59)	161 698 (59)
Nonwhite	223 081 (24)	107 829 (25)	2626 (15)	15 412 (22)	53 157 (20)
Physical disabilities	616 906 (65)	302 203 (70)	8927 (50)	42 697 (62)	152 979 (56)
Dual Medicare-Medicaid coverage	544 666 (57)	272 194 (63)	6395 (36)	35 241 (51)	126 496 (46)
Substance use disorder diagnosis (excluding opioid use disorder)	145 980 (15)	89 182 (21)	2675 (15)	9867 (14)	45 202 (17)
Opioid use disorder diagnosis	179 466 (19)	70 038 (16)	2321 (13)	8567 (12)	39 214 (14)
Outcomes					
New buprenorphine-naloxone prescriptions	78 061 (8)	33 277 (8)	1267 (7)	6117 (9)	23 722 (9)
Any buprenorphine-naloxone prescriptions	118 875 (13)	49 455 (11)	1954 (11)	9648 (14)	37 654 (14)
Any inpatient admissions	270 430 (28)	129 014 (30)	4900 (28)	15 243 (22)	76 780 (28)
Substance use disorder–related inpatient admissions	98 906 (10)	48 695 (11)	1472 (8)	5427 (8)	24 866 (9)
Any emergency department visits	434 866 (46)	213 598 (49)	7200 (40)	24 183 (35)	118 631 (44)
Substance use disorder–related emergency department visits	70 004 (7)	35 063 (8)	1008 (6)	3592 (5)	16 825 (6)
Prescription drug expenditures per month, mean (SD), $	504.6 (911.0)	471.9 (869.7)	498.6 (874.4)	558.3 (959.8)	519.9 (865.9)
Medical care expenditures per month, mean (SD), $	1132.6 (2706.5)	1157.9 (2835.0)	1125.7 (2350.9)	872.6 (2429.9)	1122.0 (2490.9)

Abbreviation: PA, prior authorization.

Before conducting formal statistical modeling to measure the association of prior authorization with use of buprenorphine-naloxone, we plotted the trends in buprenorphine-naloxone use before and after plans either removed or added prior authorization. These results show an increase (or decrease) in buprenorphine-naloxone use in the month after plans removed (or added) prior authorization for buprenorphine-naloxone (eFigure in the Supplement). We also conducted a preliminary interrupted time series analysis that only used plans that either removed or added prior authorization during the study period. These results are available in eTable 2 in the Supplement.

### Association of Prior Authorization With Use of Buprenorphine-Naloxone

Table 2 shows that removal of prior authorization was associated with an increase of 1.8 new prescriptions for buprenorphine-naloxone per plan per year (95% CI, 0.8 to 2.9 new prescriptions per plan per year) and an increase of 17.9 total prescriptions for buprenorphine-naloxone per plan per year (95% CI, 1.1 to 34.7 total prescriptions per plan per year). These changes represent an approximately 28% increase in new prescriptions and more than double the number of prescriptions per plan per year, on average. Conversely, the addition of prior authorization was associated with a decrease of 7.4 new prescriptions for buprenorphine-naloxone per month (95% CI, −10.4 to 4.4 new prescriptions per month) and a decrease of 45.9 total prescriptions for buprenorphine-naloxone prescriptions per plan per year (95% CI, −76.3 to −15.5 refill prescriptions per plan per year). These changes represent an approximately 15% decrease in new prescriptions and approximately a 60% decrease in total prescriptions per plan per year, on average. Graphs of the trends in buprenorphine-naloxone use before and after plans either removed or added prior authorization are available in the eFigure in the Supplement. Full regression results are available in eTable 3 in the Supplement.

**Table 2.** Associations of Removal or Addition of Prior Authorization With Use of Buprenorphine-Naloxone^a^

Outcome	Removal of prior authorization (n = 62 765 plan months)		Addition of prior authorization (n = 47 631 plan months)	
	Estimate, mean (95% CI)	*P* value	Estimate, mean (95% CI)	*P* value
New buprenorphine-naloxone prescriptions	1.8 (0.8-2.9)	<.001	−7.4 (−10.4 to −4.4)	<.001
Any buprenorphine-naloxone prescriptions	17.9 (1.1-34.7)	.047	−45.9 (−76.3 to −15.5)	.01

^a^ All results were estimated via generalized linear models with a log link and Poisson family, and were adjusted for demographic characteristics: mean age, percentage female, percentage dually eligible for Medicare and Medicaid, and percentage with physical disabilities. All results are marginal effects, which are interpreted as the change in the outcome. Standard errors were clustered at the plan level.

### Association of Buprenorphine-Naloxone Prescription Rates With Health Care Use

Table 3 shows that each additional buprenorphine-naloxone prescription filled was associated with 0.3 fewer inpatient admissions per plan per year (95% CI, −0.4 to −0.2 admissions per plan per year), 0.1 fewer SUD-related inpatient admissions per plan per year (95% CI, −0.2 to −0.1 admissions per plan per year), 0.7 fewer ED visits per plan per year (95% CI, −0.9 to −0.5 visits per plan per year), 0.1 fewer SUD-related ED visits per plan per year (95% CI, −0.13 to −0.03 visits per plan per year), a $3 increase in prescription drug expenditures per plan per year (95% CI, $2.3 to $3.1 per plan per year), and a $26.8 reduction in total medical care expenditures per plan per year (95% CI, −$28.8 to −$24.8 per plan per year) (all *P* < .001). These changes represent an approximately 0.3% decrease in inpatient admissions, an approximately 0.3% decrease in SUD-related inpatient admissions, a 0.4% decrease in ED visits, a 0.4% decrease in SUD-related ED visits, a 0.5% increase in prescription drug expenditures, and a 2.4% decrease in total nondrug health care expenditures. Full regression results are reported in eTable 4 in the Supplement.

**Table 3.** Association of the Number of Any Buprenorphine-Naloxone Prescriptions With Health Care Outcomes^a^

Outcome	Any buprenorphine-naloxone prescriptions (N = 110 396 plan months)	
	Estimate, mean (95% CI)	*P* value
All-cause inpatient admissions	−0.3 (−0.4 to −0.2)	<.001
Substance use disorder–related inpatient admissions	−0.1 (−0.2 to −0.1)	<.001
All-cause emergency department visits	−0.7 (−0.9 to −0.5)	<.001
Substance use disorder–related emergency department visits	−0.1 (−0.13 to −0.03)	<.001
Prescription drug expenditures, $	2.7 (2.3 to 3.1)	<.001
Nondrug expenditures, $	−26.8 (−28.8 to −24.8)	<.001

^a^ All results were estimated via generalized linear models. The count outcomes (all-cause inpatient admissions, substance use disorder–related inpatient admissions, all-cause emergency department visits, and substance use disorder–related emergency department visits) were estimated with a log link and Poisson family. The expenditure outcomes (prescription drug expenditures and nondrug expenditures) were estimated with a log link and γ family. All models were adjusted for demographic characteristics: mean age, percentage female, percentage dually eligible for Medicare and Medicaid, and percentage with physical disabilities. All results are marginal effects, which are interpreted as the change in the outcome. Standard errors were clustered at the plan level.

### Association of Prior Authorization With Health Care Outcomes

Table 4 shows that after combining the results from Table 2 with the results from Table 3, the removal of prior authorization was associated with 5.7 fewer inpatient admissions per plan per year (95% CI, −12.1 to −0.3 admissions per plan per year), 2.0 fewer SUD-related inpatient admissions per plan per year (95% CI, −4.3 to −0.1 admissions per plan per year), 12.6 fewer ED visits per plan per year (95% CI, −25.9 to −0.5 ED visits per plan per year), 1.4 fewer SUD-related ED visits per plan per year (95% CI, −3.2 to −0.1 ED visits per plan per year), a $48.7 increase in prescription drug expenditures per plan per year (95% CI, $3.1 to $96.0 per plan per year), and a $479.2 decrease in total nondrug health care expenditures per plan per year (95% CI, −$942.7 to −$21.1 per plan per year). These changes represent an approximately 24% decrease in inpatient admissions, an approximately 28% decrease in SUD-related inpatient admissions, a 36% decrease in ED visits, and a 29% decrease in SUD-related ED visits. Table 4 also shows that after combining the results in Table 2 with the results in Table 3, the addition of prior authorization was associated with 14.7 more inpatient admissions per plan per year (95% CI, 4.6 to 27.2 admissions per plan per year), 5.1 more SUD-related inpatient admissions per plan per year (95% CI, 1.5 to 9.8 admissions per plan per year), 32.2 more ED visits per plan per year (95% CI, 10.2 to 57.6 ED visits per plan per year), 3.6 more SUD-related ED visits per plan per year (95% CI, 0.8 to 7.5 ED visits per plan per year), a $124.7 decrease in prescription drug expenditures per plan per year (95% CI, −$214.2 to −$40.6 per plan per year), and a $1236.9 increase in total nondrug health care expenditures per plan per year (95% CI, $434.2 to $2055.0 per plan per year). These changes represent an approximately 9% increase in inpatient admissions, a 10% increase in SUD-related inpatient admissions, a 13% increase in ED visits, and a 10% increase in SUD-related ED visits.

**Table 4.** Association of Removal or Addition of Prior Authorization With Health Care Outcomes^a^

Outcome	Removal of prior authorization: plans that removed prior authorization (n = 62 765 plan months)		Addition of prior authorization: plans that added prior authorization (n = 47 631 plan months)	
	Estimate, mean (95% CI)	*P* value	Estimate, mean (95% CI)	*P* value
All-cause inpatient admissions	−5.7 (−12.1 to −0.3)	.04	14.7 (4.6 to 27.2)	.003
Substance use disorder–related inpatient admissions	−2.0 (−4.3 to −0.1)	.04	5.1 (1.5 to 9.8)	.004
All-cause emergency department visits	−12.6 (−25.9 to −0.5)	.04	32.2 (10.2 to 57.6)	.004
Substance use disorder–related emergency department visits	−1.4 (−3.2 to −0.1)	.04	3.6 (0.8 to 7.5)	.005
Prescription drug expenditures, $	48.7 (3.1 to 96.0)	.04	−124.7 (−214.2 to −40.6)	.003
Nondrug expenditures, $	−479.2 (−942.7 to −21.1)	.04	1236.9 (434.2 to 2055.0)	.003

^a^ The results in this table multiplicatively combine the results from Table 2 and Table 3. All results are marginal effects, which are interpreted as the change in the outcome. Standard errors were clustered at the plan level. Confidence intervals and *P* values were obtained using a generalized Hausman test.

## Discussion

The US is implementing a variety of policies to reduce deaths from OUD. This study found that expanding access to buprenorphine-naloxone, by removing prior authorization restrictions, is associated with increased use of buprenorphine-naloxone and improved SUD-related health care outcomes. Considering the observed increasing rates of OUD in the Medicare population, and previously documented underutilization of medications for the treatment of OUD in the Medicare population, these results suggest that improving access and uptake of these medications could improve the health care outcomes of hundreds of thousands of Medicare beneficiaries.

Prior authorization policies are used to encourage effective and cost-effective use of medications. However, we found that rather than encouraging more-effective medication use, prior authorization policies for buprenorphine-naloxone were associated with decreased use of buprenorphine-naloxone overall. Furthermore, we found that lower buprenorphine-naloxone use was associated with higher hospitalization and ED visit rates. These findings suggest that the costs of using prior authorization for buprenorphine-naltrexone may outweigh potential economic benefits.

### Limitations

This study is limited in that we used hospital and ED visits as a proxy for negative health outcomes. Further research could examine mortality outcomes among Medicare beneficiaries. Second, we did not capture potential negative outcomes of expanding OUD medications on other populations, such as children who accidently ingest buprenorphine-naloxone or diversion to other populations for recreational use. In addition, the study was limited to Medicare beneficiaries and should be replicated with Medicaid and privately insured populations.

## Conclusions

Prior authorization is commonly used for buprenorphine-naloxone because of concerns regarding costs and diversion. These findings suggest that these concerns may be unfounded and that requiring prior authorization before one can access a buprenorphine-naloxone product may be more harmful than beneficial.

## Notes

Supplement. **eTable 1.** Coverage of Buprenorphine-Naloxone Products During Study Period **eFigure.** Trends in the Number of Buprenorphine-Naloxone Prescriptions in the 12 Months Before and After Plans Removed or Added Prior Authorization **eTable 2.** Regression Coefficients From Analyses of the Association Between Prior Authorization and Use of Buprenorphine-Naloxone **eTable 3.** Regression Coefficients From Equation 2 Analyses of the Association Between Use of Buprenorphine-Naloxone and Health Care Outcomes **eTable 4.** Results From a Poisson Interrupted Time Series Analysis
