# Supplementary material for: Association of Formulary Prior Authorization Policies With Buprenorphine-Naloxone Prescriptions and Hospital and Emergency Department Use Among Medicare Beneficiaries
Source: JAMA Netw Open. 2020 Apr 20;3(4):e203132. doi: 10.1001/jamanetworkopen.2020.3132 (PMC7171554; doi:10.1001/jamanetworkopen.2020.3132)
Supplement: Supplement. — eTable 1. Coverage of Buprenorphine-Naloxone Products During Study Period eFigure. Trends in the Number of Buprenorphine-Naloxone Prescriptions in the 12 Months Before and After Plans Removed or Added Prior Authorization eTable 2. Regression Coefficients From Analyses of the Association Between Prior Authorization and Use of Buprenorphine-Naloxone eTable 3. Regression Coefficients From Equation 2 Analyses of the Association Between Use of Buprenorphine-Naloxone and Health Care Outcomes eTable 4. Results From a Poisson Interrupted Time Series Analysis [file jamanetwopen-3-e203132-s001.pdf]

Supplementary Online Content

Mark TL, Parish WJ, Zarkin GA. Association of formulary prior authorization policies with buprenorphine-naloxone prescriptions and hospital and emergency department use among Medicare beneficiaries. *JAMA Netw Open*. 2020;3(4):e203132. doi:10.1001/jamanetworkopen.2020.3132

eTable 1. Coverage of Buprenorphine-Naloxone Products During Study Period

eFigure. Trends in the Number of Buprenorphine-Naloxone Prescriptions in the 12 Months Before and After Plans Removed or Added Prior Authorization

eTable 2. Regression Coefficients From Analyses of the Association Between Prior Authorization and Use of Buprenorphine-Naloxone

eTable 3. Regression Coefficients From Equation 2 Analyses of the Association Between Use of Buprenorphine-Naloxone and Health Care Outcomes

eTable 4. Results From a Poisson Interrupted Time Series Analysis

This supplementary material has been provided by the authors to give readers additional information about their work.

© 2020 Mark TL et al. *JAMA Network Open.*

eTable 1 shows that nearly 100% of plans covered at least one brand name buprenorphine-naloxone product in all years between 2012 and 2017. Starting in 2013, a new generic buprenorphine-naloxone product became available for the first time, and approximately two thirds of plans began providing coverage for this product.

eTable 1. Coverage of Buprenorphine-Naloxone Products During Study Period

Year	% That Cover Brand Name Buprenorphine-Naloxone	% That Cover Generic Buprenorphine-Naloxone
2012	100%	0%
2013	99%	71%
2014	98%	67%
2015	98%	65%
2016	98%	72%
2017	97%	75%

Table reports percent of plans that cover buprenorphine-naloxone products by brand name or generic status.

© 2020 Mark TL et al. *JAMA Network Open.*

eFigure. Trends in the Number of Buprenoprhine-Naloxone Prescriptions in the 12 Months Before and After Plans Removed or Added Prior Authorization

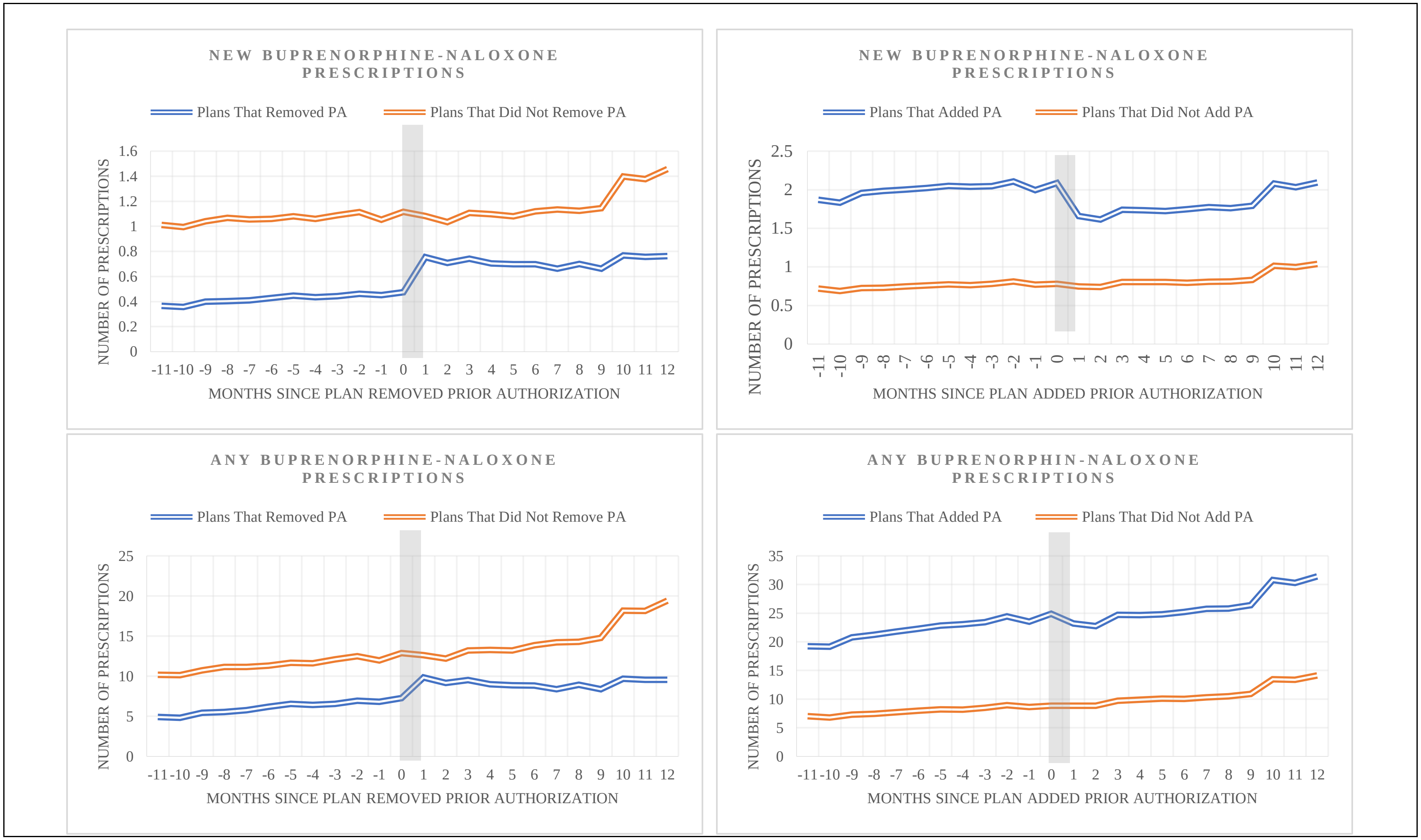

Notes. eFigure shows the pre- and post-formulary change trends in buprenorphine-naloxone prescription fills.

PA, prior authorization. The gray bar highlights the period during which it was hypothesized that there would be an increase/decrease in the number of prescriptions.

© 2020 Mark TL et al. *JAMA Network Open.*

eTables 2 and 3 show the raw regression coefficients from all regression specifications reported in this paper. The raw regression coefficients have not been adjusted to account for the fact that the outcomes were logged, and accordingly, the results in these tables are not equivalent to the results in the tables in the main body of the paper.

eTable 2. Regression Coefficients From Analyses of the Association Between Prior Authorization and Use of Buprenorphine-Naloxone

**Covariate**	**Plans That Removed Prior Authorization**		**Plans That Added Prior Authorization**	
	**# of Persons with New Prescriptions (in logs)**	**# of Persons with Any Prescriptions (in logs)**	**# of Persons with New Prescriptions (in logs)**	**# of Persons with Any Prescriptions (in logs)**
	**Coef. (SE)**	**Coef. (SE)**	**Coef. (SE)**	**Coef. (SE)**
N	62,765	62,765	47,631	47,631
Addition or Removal of Prior Authorization	0.2297*	0.2147**	-0.3051***	-0.1370**
	(0.1130)	(0.0772)	(0.0469)	(0.0454)
Month	-0.0054***	0.0050***	-0.0039	0.0084**
	(0.0013)	(0.0004)	(0.0022)	(0.0025)
Addition or Removal of Prior Authorization by Month	-2.7166***	-6.8891***	-3.6925***	-4.4864***
	(0.5475)	(0.1363)	(0.8421)	(0.9552)
Added or Removed Prior Authorization	0.6248***	-0.8555***	0.7154*	0.8547**
	(0.1871)	(0.0687)	(0.2910)	(0.3073)
Average Age	1.9827***	11.0024***	0.6210	2.1129*
	(0.5819)	(0.1527)	(0.9892)	(1.0409)
% Female	0.2297*	0.2147**	-0.3051***	-0.1370**
	(0.1130)	(0.0772)	(0.0469)	(0.0454)
% Dually Eligible	-0.0054***	0.0050***	-0.0039	0.0084**
	(0.0013)	(0.0004)	(0.0022)	(0.0025)
% Disabled	-0.0110	-0.0505***	-0.0075*	-0.0130***
	(0.0066)	(0.0029)	(0.0029)	(0.0030)

Notes. Standard errors were clustered at the plan-level.

© 2020 Mark TL et al. *JAMA Network Open.*

eTable 3. Regression Coefficients From Equation 2 Analyses of the Association Between Use of Buprenorphine-Naloxone and Health Care Outcomes

Covariate	# of Persons with IP Admissions (in logs)	# of Persons With SUD-Related IP Admissions (in logs)	# of Persons with ED Visits (in logs)	# of Persons with SUD-Related ED Visits (in logs)	Average Prescription Spending	Average Nondrug Health Care Spending
	**Coef. (SE)**	**Coef. (SE)**	**Coef. (SE)**	**Coef. (SE)**	**Coef. (SE)**	**Coef. (SE)**
N	110,396	110,396	110,396	110,396	110,396	110,396
# of Persons Filling Any Prescriptions	-0.0010***	-0.0011***	-0.0012***	-0.0012**	0.2327***	-1.5298***
	(0.0002)	(0.0003)	(0.0002)	(0.0004)	(0.0144)	(0.0647)
Month	-0.0159***	-0.0147***	-0.0164***	-0.0142***	3.1295***	3.9798***
	(0.0006)	(0.0007)	(0.0006)	(0.0008)	(0.0393)	(0.1122)
Average Age	0.0044	-0.0068	-0.0295*	-0.0253	12.9649***	-99.3975***
	(0.0126)	(0.0141)	(0.0116)	(0.0158)	(0.5759)	(1.3623)
% Female	1.0634**	-1.6167***	2.6549***	-1.5561**	341.5696***	1334.8540***
	(0.3765)	(0.4664)	(0.3782)	(0.5952)	(13.1704)	(27.8552)
% Dually Eligible	0.1989	0.5808**	0.2281	0.8084**	281.0899***	-533.5401***
	(0.1596)	(0.1901)	(0.1500)	(0.2693)	(4.6554)	(9.4468)
% Disabled	-0.6032	-0.6791	-0.6254*	-0.7807	328.0114***	292.6668***
	(0.3523)	(0.4063)	(0.3138)	(0.4286)	(15.2903)	(1.8940)

Notes. Standard errors were clustered at the plan-level.

© 2020 Mark TL et al. *JAMA Network Open.*

eTable 4 shows the results from an interrupted time series analysis that does not include a comparison group to control for secular trends in the outcome. This analysis also uses a generalized linear model with log link and Poisson family.

eTable 4. Results From a Poisson Interrupted Time Series Analysis

**Outcome**	**Removal of Prior Authorization (N=62765 Plan Months)**		**Addition of Prior Authorization (N=47,631 Plan Months)**	
	**Estimate (95% CI)**	***P* Value**	**Estimate (95% CI)**	***P* Value**
% Change in New Buprenorphine-Naloxone Prescriptions	32.7% (16.3%, 51.4%)	<0.001	−30.5% (−37.0%, −23.3%)	<0.001
% Change in Any Buprenorphine-Naloxone Prescriptions	24.2% (10.5%, 39.6%)	<0.001	−22.4% (−28.7%, −15.5%)	<0.001

Abbreviations: CI, confidence interval.
All results are adjusted for demographic characteristics: average age, % female, % dually eligible for Medicare and Medicaid, and % disabled.
All estimates have been adjusted from the raw coefficients with the following formula: (exp(b) – 1) *100. Estimates are interpreted as the percentage change in the outcome.
Standard errors were clustered at the plan-level.

© 2020 Mark TL et al. *JAMA Network Open.*
